# Bullous Hemorrhagic Dermatosis: A Rare Benign Cutaneous Complication of Low-Molecular-Weight Heparin

**DOI:** 10.7759/cureus.31173

**Published:** 2022-11-06

**Authors:** Arnab Choudhury, Nonita Thokchom, Vikram Jain, Mukesh Bairwa

**Affiliations:** 1 Internal Medicine, All India Institute of Medical Sciences, Rishikesh, Rishikesh, IND

**Keywords:** ecchymosis, histopathology, hypersensitivity, enoxaparin, bullous haemorrhagic dermatosis

## Abstract

Enoxaparin-mediated bullous hemorrhagic dermatosis (BHD) is one of the rare side effects during prophylaxis of enoxaparin for various thromboembolic events. We report a case of a 74-year-old female with multiple comorbidities who developed BHD at a distant site from subcutaneous delivery of enoxaparin. Histopathological analysis confirmed the diagnosis of BHD. Discontinuation of enoxaparin resulted in the gradual resolution of the bullae formation, and the patient was started on novel oral anticoagulation with apixaban. The usual cutaneous adverse effects of enoxaparin include maculopapular rash, pruritus, skin necrosis, eczematous dermatitis, and rarely bullous hemorrhagic dermatosis. This hemorrhagic bullae dermatosis at a distant site from the administration is a relatively rare and benign side effect of enoxaparin which is an under-recognized complication of low-molecular-weight heparin.

## Introduction

Heparin is the most widely used form of anticoagulation in primary and secondary prophylaxis of thromboembolic disorders, including acute coronary syndrome, deep vein thrombosis, pulmonary thromboembolism, and autoimmune disorders with an increased risk of thrombosis. Currently, two forms of heparin preparation are available - unfractionated heparins (UFHs) and low-molecular-weight heparins (LMWHs) [1]. The various adverse effects due to prophylactic or therapeutic administration of heparin are heparin-induced thrombocytopenia (HIT) and various forms of cutaneous reactions. These cutaneous adverse effects have local or generalized manifestations and range from allergic reactions (urticaria, erythema, or dermatitis) to more severe reactions like intradermal microvascular thrombosis as a result of HIT [2]. However, infrequently rare cutaneous complications in the form of bullous hemorrhagic dermatosis (BHD) are also reported in the literature. The first case of BHD was reported in 2004 by Dyson et al. [3] as a new clinical manifestation of cutaneous side effects of LMWHs at a site distant from the injection site. Histological analysis revealed the presence of intraepidermal blisters filled with red blood cells [4]. The exact mechanism of BHD is not clearly elucidated, but the proposed mechanism encompasses delayed hypersensitivity, high doses of anticoagulant use, and antibodies towards heparin-platelet factor 4 (HPF-4) [5]. To date, less than 20 cases have been reported worldwide in various sources of literature. Here we report a similar case to highlight the occurrence of this rare side effect after the administration of low-molecular-weight heparin.

## Case presentation

An elderly female in her mid 70's with multiple comorbidities, including morbid obesity, chronic obstructive pulmonary disease, hypertension, type 2 diabetes mellitus, and coronary artery disease, on medical treatment, developed a gradual worsening of shortness of breath from New York Heart Association (NHYA) grade 2 to grade 3 with a history of orthopnea and paroxysmal nocturnal dyspnoea (PND), with bilateral painless symmetrical pitting edema and facial puffiness.

**Figure 1:**
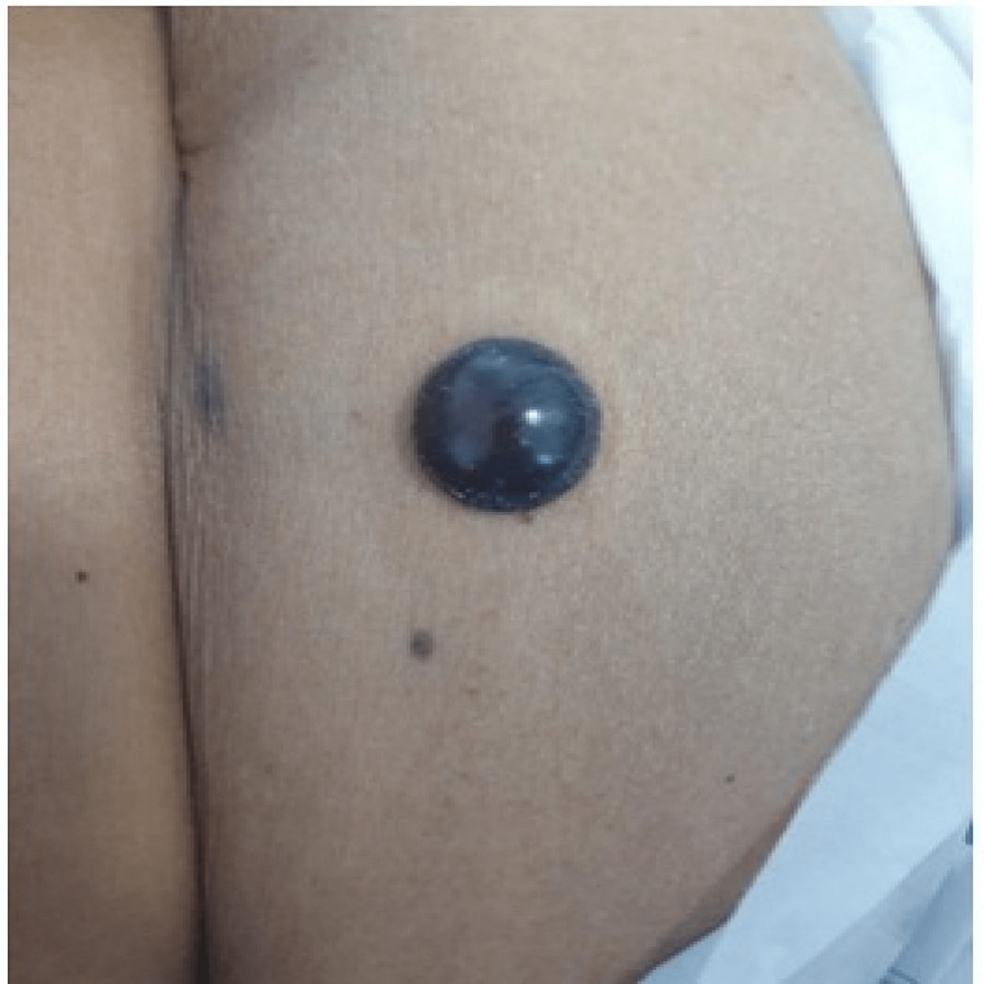
Day 6 of enoxaparin administration A black bullae eruption of 2x3 cm in size was noted over the periumbilical area.

**Figure 2:**
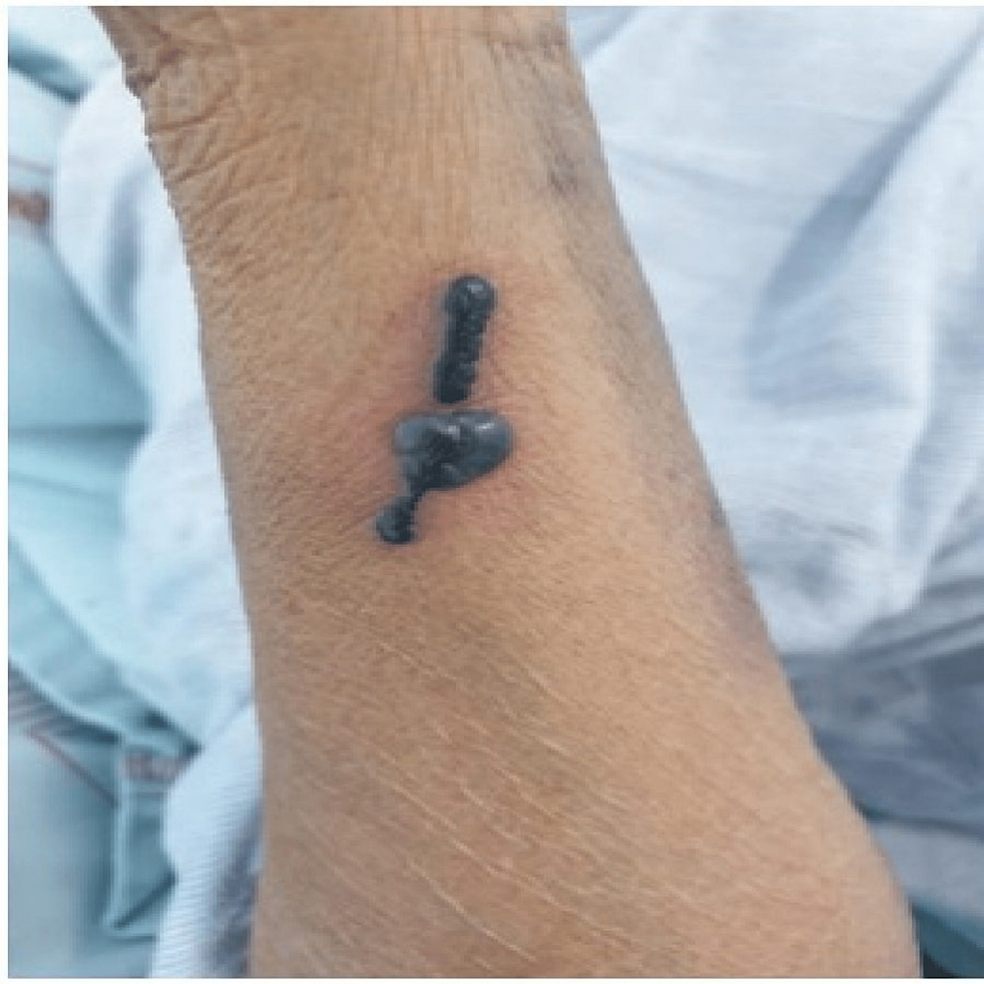
Day 8 of enoxaparin administration Blackish bullae were noted on the lateral aspect of the right forearm.

**Figure 3:**
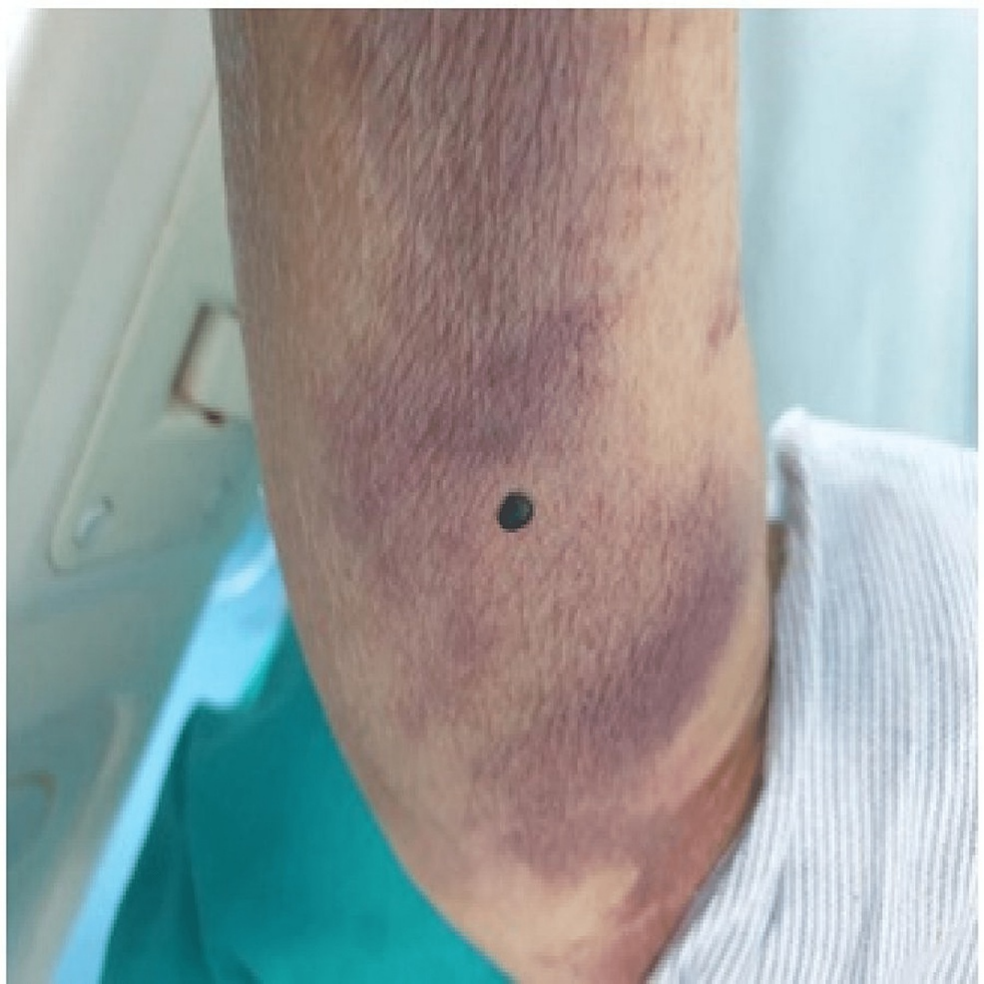
Day 9 of enoxaparin administration Blackish bullae eruption 1x1 cm in size on the left upper medial forearm.

At presentation, her ECG showed Mobitz type 2 atrioventricular block and hyperkalemia, which were promptly corrected medically, after which the heart block resolved. Transthoracic echocardiography showed atrial septal defect with Eisenmengerization along with heart failure with reduced left ventricular ejection fraction (LVEF-35%) and pulmonary arterial hypertension. The patient underwent CT pulmonary angiography (CTPA), which showed multiple intraluminal filling defects in bilateral subsegmental pulmonary arteries, suggestive of subsegmental pulmonary embolism. Subsequently, low-molecular-weight heparin (enoxaparin) in a dose of 1 mg/kg twice a day subcutaneously was started. Her vital parameters and general condition improved subsequently. After six days of initiating enoxaparin, she developed a blackish bullous eruption (Figure 1) without any signs of inflammation or cellulitis over the periumbilical area where enoxaparin was administered. After three days, similar lesions were also noted on two distal locations, one on the right forearm (Figure 2) and one on the left forearm (Figure 3).

**Figure 4:**
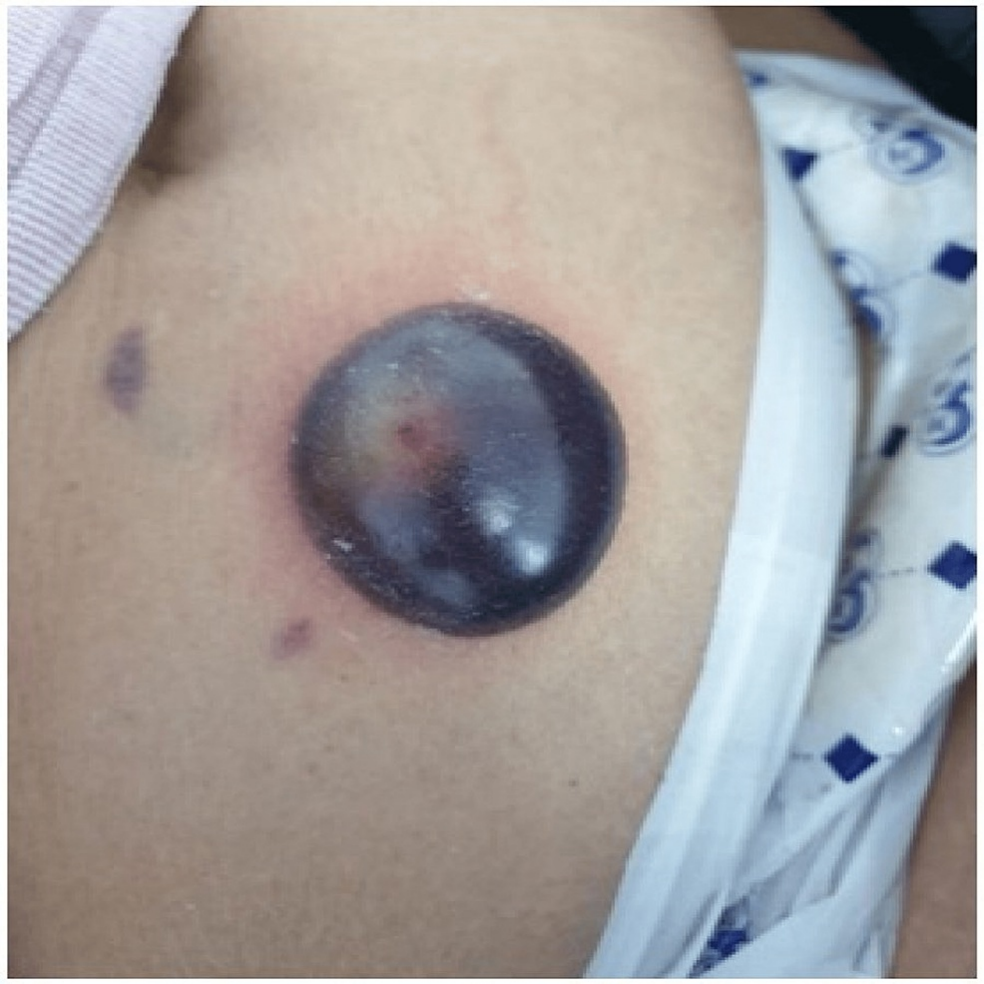
Day 14 of enoxaparin administration Increase in the size of periumbilical bullae 4x5 cm in size.

Five days after the onset of the first lesion, the lesion developed into large hemorrhagic bullae measuring 4x5 cm (Figure 4). 

Investigations

**Figure 5:**
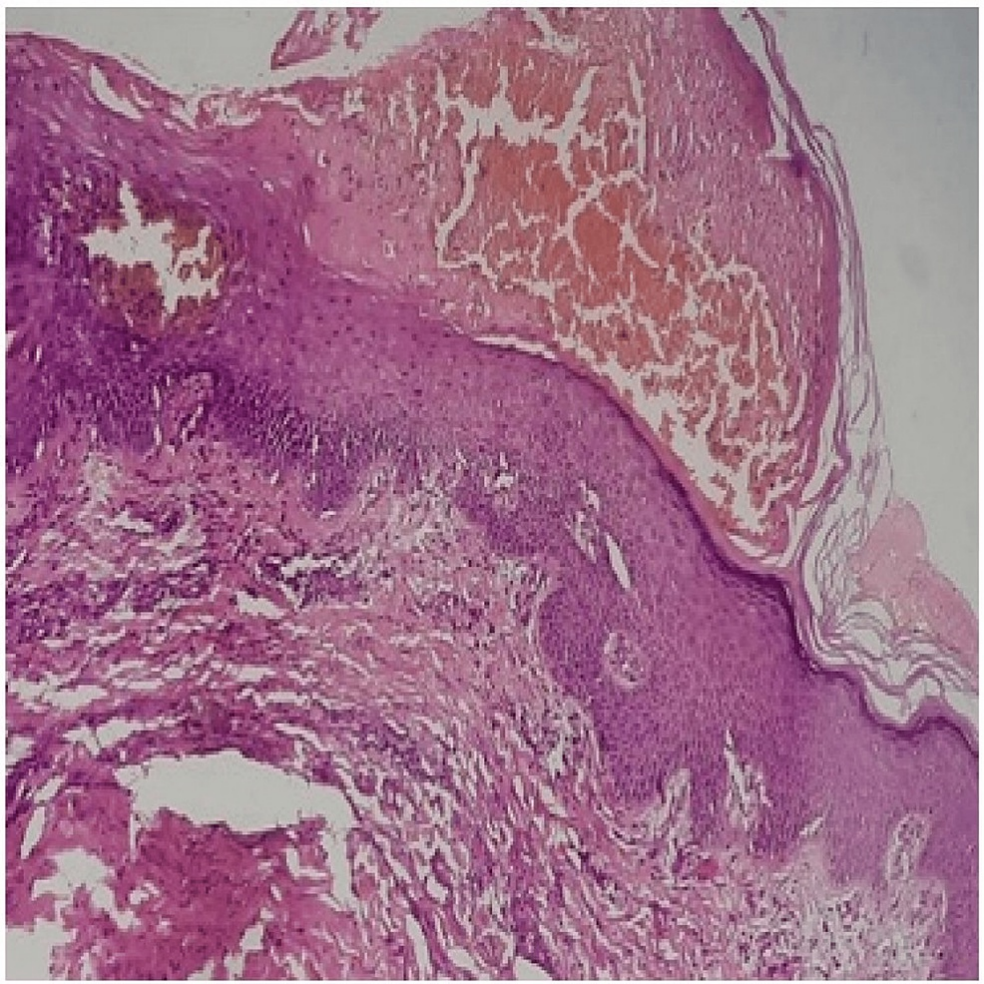
Skin biopsy showing intracorneal blister filled with red blood cells, scattered neutrophils, plasma cells, and few eosinophils The dermis shows mild chronic inflammation. Features suggestive of drug-induced bullous dermatosis.

Her coagulation profile and platelets were within normal range. Platelet function testing was not considered, and thrombophilia workup for hypercoagulable states was deferred. After ruling out any infective complications, the diagnosis of heparin-induced hemorrhagic bullous dermatosis was made on the basis of clinical examination and confirmed by histopathological examination (Figure 5). 

Differential diagnosis

Heparin-induced skin necrosis and atypical bullous pemphigoid were the alternative diagnoses that were considered initially, but a histopathological report confirmed it to be a case of bullous hemorrhagic dermatosis likely due to adverse drug reaction.

Treatment and outcome

Enoxaparin was discontinued, and the patient was switched to oral anticoagulation in the form of apixaban for pulmonary thromboembolism. The lesions gradually diminished in size and intensity with complete resolution subsequently.

The skin lesions healed within four weeks of stopping the offending drug, with no recurrence of bullae after starting apixaban. 

## Discussion

BHD is a rare side effect of subcutaneously administered low-molecular-weight heparin. BHD usually manifests on an average within seven days after initiation of heparin therapy; however, some reports show that it may occur as early as on the first day and as late as after six to nine months of heparin administration in patients requiring prolonged subcutaneous anticoagulation [4]. In the majority of cases, the blisters occur at extremities distant from the site of injection and rarely over the trunk, face, and neck. Most of the BHD lesions are caused by enoxaparin and rarely by other types of LMWHs such as dalteparin, tinzaparin, bemiparin, unfractionated heparin, and fondaparinux. The present case demonstrates the occurrence of this lesion on the sixth day of enoxaparin administration at the site of injection, followed by similar lesions in areas distant from the site of drug delivery. 

The BHD lesions usually range from small to large size, dark hemorrhagic blisters, albeit they resemble clinically relevant entities of subepidermal blisters, such as necrotic skin or bullous vasculitis and pemphigoid and these lesions are characterized by urticarial plaques with pain [5]. However, our patient had not shown any of these manifestations. To confirm the clinical suspicion and rule out other causes of epidermal skin blisters and bullous lesions, a skin biopsy is mandatory to confirm the diagnosis.

BHD histopathological morphology reveals the presence of intraepidermal blisters filled with red blood cells. Also, lesions should generally be devoid of microthrombi in the vessel, which is indicative of skin necrosis or vascular lesions suggestive of vasculitis. There was no evidence of any autoimmune etiology for the bullous formation, as evident on negative direct and indirect immunofluorescence assay, except in a patient presenting with bullous pemphigoid [6]. Our case also displayed intraepidermal blisters filled with red blood cells, scattered neutrophils, plasma cells, and a few eosinophils, which are similar to the earlier reported cases [4,5].

The etiology of BHD is not very clear, and one of the prime mechanisms is a hypersensitivity reaction to the offending drug, which is evidenced by the presence of eosinophils in histology [5]. To support this, the lesions in our patient also revealed the presence of eosinophils during the histological examination, suggestive of likely drug hypersensitivity. Studies also state that co-administration of anti-platelet or warfarin, along with heparin, is also associated with BHD [7]. However, the coagulation profile and platelet counts were normal in our case at the time of the appearance of the lesion, which is similar to other reported cases. In one case, it is reported as the involvement of heparin platelet factor 4 (HPF-4) antibody, but HPF-4 antibody is usually observed in heparin-induced skin necrosis at the injection site. Further, histological analysis reveals the presence of microthrombi in the vessels [7].

To date, there is no definitive treatment for BHD. In a study done by Uceda-Martin et al. among 33 cases, the bullous lesions disappeared after stopping heparin treatment, with a mean time to resolution of 12.7±8.4 days [8]. However, in some severe cases, steroid therapy can be considered for the resolution of the lesions if there are concomitant bullous pemphigoid lesions [6]. Some clinicians reported similar cases, but even when heparin was kept administered for some time, the lesions improved. This improvement may be due to the natural course of this disease which is typically self-limiting [2]. Our case is unusual, as, after the sixth day of subcutaneous injection of enoxaparin, gradual progressive painless hemorrhagic bullae developed over the site of injection without any inflammation, histopathologically showing intraepithelial blisters filled with red blood cells, scattered neutrophils, plasma cells, and a few eosinophils. Based on the present case report, we conclude that bullous hemorrhagic dermatosis is a rare adverse cutaneous effect of low-molecular-weight heparin that should be considered in patients who have recently started heparin therapy and who have suggestive clinical and histopathologic findings.

## Conclusions

Bullous hemorrhagic dermatosis is an unusual adverse reaction due to the administration of low-molecular-weight heparin, which is unusual but completely treatable and curable. Careful monitoring is required during heparin administration since these cutaneous reactions occur at any point in time, irrespective of the duration of anticoagulation therapy. Switching over to alternative oral anticoagulants and prompt discontinuation of subcutaneous low-molecular-weight heparin with local care of the blisters and surrounding skin appears to be the only treatment modality in such cases.
